# Revisiting the calpain hypothesis of learning and memory 40 years later

**DOI:** 10.3389/fnmol.2024.1337850

**Published:** 2024-02-01

**Authors:** Michel Baudry, Xiaoning Bi

**Affiliations:** Western University of Health Sciences, Pomona, CA, United States

**Keywords:** calpain, Long-Term Potentiation, hippocampus, learning and memory, neurodegeneration

## Abstract

In 1984, Gary Lynch and Michel Baudry published in *Science* a novel biochemical hypothesis for learning and memory, in which they postulated that the calcium-dependent protease, calpain, played a critical role in regulating synaptic properties and the distribution of glutamate receptors, thereby participating in memory formation in hippocampus. Over the following 40 years, much work has been done to refine this hypothesis and to provide convincing arguments supporting what was viewed at the time as a simplistic view of synaptic biochemistry. We have now demonstrated that the two major calpain isoforms in the brain, calpain-1 and calpain-2, execute opposite functions in both synaptic plasticity/learning and memory and in neuroprotection/neurodegeneration. Thus, calpain-1 activation is required for triggering long-term potentiation (LTP) of synaptic transmission and learning of episodic memory, while calpain-2 activation limits the magnitude of LTP and the extent of learning. On the other hand, calpain-1 is neuroprotective while calpain-2 is neurodegenerative, and its prolonged activation following various types of brain insults leads to neurodegeneration. The signaling pathways responsible for these functions have been identified and involve local protein synthesis, cytoskeletal regulation, and regulation of glutamate receptors. Human families with mutations in calpain-1 have been reported to have impairment in motor and cognitive functions. Selective calpain-2 inhibitors have been synthesized and clinical studies to test their potential use to treat disorders associated with acute neuronal damage, such as traumatic brain injury, are being planned. This review will illustrate the long and difficult journey to validate a bold hypothesis.

## 1 Introduction

Despite over a hundred years of research, the cellular/molecular mechanisms underlying learning and memory are still not completely understood. Many hypotheses have been proposed, but there is no consensus for any of these. The majority of the models attempting to account for the features of learning and memory are based on the existence of synaptic plasticity, which represents activity-dependent modifications of synaptic efficacy at both excitatory and inhibitory synapses. One of the prevalent forms of synaptic plasticity is referred to as Long-Term Potentiation (LTP) of synaptic transmission at excitatory synapses. LTP was first discovered in hippocampus by Bliss and Lomo (1973) and has been the subject of intense investigation ever since. LTP has many features that match the features of learning; it is optimally and rapidly elicited by brief trains of electrical stimulation with short bursts of high frequency repeated at theta frequency, the brain rhythm associated with exploration; it can last a very long time [as long as can be recorded in living animals] and has associative properties. It is prevalent in the various circuitries of the hippocampus, the brain structure that is critical for memory formation. Pharmacological or genetic manipulations that affect LTP have similar effects on learning and memory (Lynch and Baudry, 1988). It has been therefore widely accepted that LTP plays a very significant role in storing information in hippocampus and other brain circuitries that exhibit this phenomenon. Understanding learning and memory has thus been reduced to understand the mechanisms responsible for eliciting and maintaining LTP at excitatory synapses.

Lynch and Baudry (1984) proposed the hypothesis that activation of the calcium-dependent protease, calpain, following brief trains of high frequency stimulation, resulted in an increase in the number of glutamate receptors at excitatory synapses in hippocampus, as well as in a modification of the structure of the dendritic spines, leading to more stable spines. It took 30 years to provide convincing evidence to support and refine this hypothesis, and the last 10 years have brought us closer to test it in non-human primates and in humans. This review will summarize the various findings we and others have collected during these 40 years, which have sometimes refuted, but ultimately validated our initial hypothesis.

## 2 The early years: 1979–1985

It all started by attempting to study glutamate receptors with ^3^H-glutamate as a ligand to label the receptors in synaptic membranes from hippocampus. A sodium-independent binding site with properties suggesting that it was a glutamate receptor was identified (Baudry and Lynch, 1979b). It was further observed that this glutamate binding site was regulated by cations, and that divalent cations increased glutamate binding (Baudry and Lynch, 1979a). Interestingly, preincubating synaptic membranes with calcium also resulted in an increase in binding. Using a variety of pharmacological tools, this effect was shown to be due to the activation by calcium of a protease (Baudry and Lynch, 1980b). The only neutral calcium-dependent protease in the brain had been identified in Guroff (1964) and named calpain in Murakami et al. (1981). At the time, there were 2 forms of calpain in the brain, calpain-1 and calpain-2, which differed by the levels of calcium required for activation *in vitro* (Yoshimura et al., 1983), with calpain-1 activated by micromolar calcium concentration, while calpain-2 required millimolar calcium concentration. The presence of calpain-1 in synaptic membranes was confirmed and a preferred substrate for this protease (Siman et al., 1983), the cytoskeletal spectrin, was also identified in synaptic membranes (Siman et al., 1984). This was particularly interesting, as spectrin was known to form a meshwork underneath the plasma membrane and to participate in the regulation of cell shape and motility. During this period, it was also discovered that LTP induction required an influx of calcium in the postsynaptic neuron (Lynch et al., 1983). The initial hypothesis therefore stated that calcium-induced breakdown of spectrin was responsible for increasing the number of glutamate receptors in synaptic membranes, which could account for the LTP phenomenon. In the model, spectrin breakdown also allowed for a remodeling of synaptic membranes and the insertion of new glutamate receptors or the unmasking of occluded receptors. This hypothesis was published in Science in 1984, under the title “The biochemistry of memory: A new and specific hypothesis”(Lynch and Baudry, 1984). Naively, Gary Lynch and Michel Baudry thought that the scientific community would follow up, and either provide supporting evidence for it or conversely disprove their hypothesis. Surprisingly, nobody seemed to care, and the hypothesis was generally ignored and dismissed as a “fantasy” by the bulk of the leaders in the field.

## 3 The higher one climbs, the harder the fall

As is often the case in science, initial findings are followed by newer ones that contradict the interpretation of the previous results. One of the key components of the hypothesis was the underlying assumption that the binding sites labeled by ^3^H-glutamate in synaptic membranes represented a subtype of glutamate receptors. Unfortunately, this turned out to be not true. One of the postdocs in Gary’s lab performed thorough and exhaustive experiments to demonstrate that this binding represented in fact glutamate sequestration mediated by a sodium-chloride exchange in vesicles present in synaptic membrane preparations (Kessler et al., 1987). This indeed constituted a major blow to the initial hypothesis since it eliminated the notion of calpain-mediated changes in glutamate receptors. In order to salvage it, Lynch and Baudry proposed that calpain activation could still participate in the structural reorganization of dendritic spines (Lynch and Baudry, 1987). Several laboratories including Gary’s lab, found that LTP induction was associated with morphological changes in the structures of dendritic spines in field CA1 of hippocampus (Lee et al., 1980; Fifkova and Anderson, 1981; Desmond and Levy, 1983; Chang and Greenough, 1984; Baudry and Bi, 2013; Lynch and Gall, 2013; Briz et al., 2015; Gall et al., 2023). Thus, spines appeared to change from elliptical to spherical. They were therefore proposing that calpain-mediated spectrin breakdown could be responsible for this structural modification, which could have several important implications. First, spherical spines could be more stable than elliptical ones, which could account for the long duration of potentiation. Second, a spherical spine would have a wider postsynaptic density than an elliptical spine, which could account for a larger number of AMPA receptors and enhanced synaptic transmission. It was then argued that calpain-mediated increase in ^3^H-glutamate transport could be the result of such structural reorganization of membrane structures (Lynch and Baudry, 1987). But obviously, this was mostly window-dressing, and different approaches were needed to further confirm the hypothesis, which contained enough compelling features to make it difficult to abandon.

In particular, administration of the calpain inhibitor leupeptin in rats was found to block LTP in field CA1 (Oliver et al., 1989). It also resulted in a selective impairment of learning in the 8-arm maze in rats (Staubli et al., 1984, Morris et al., 1987). These results therefore provided strong support for the notion that calpain activation was playing a critical role in both LTP and spatial learning. During this period of time, the use of molecular biology and cloning techniques as well as the availability of novel pharmacological compounds synthesized by Jeff Watkins provided strong evidence for the existence of multiple types of glutamate receptors (Watkins et al., 1990, Hollmann and Heinemann, 1994). Thus, three types of ionotropic glutamate receptors were identified, the AMPA receptors, the NMDA receptors and the Kainate receptors. A collaboration between the Lynch’s lab and the lab of Richard Morris confirmed that blocking the NMDA receptors with an antagonist, AP5, prevented LTP in rat hippocampus, and produced a selective impairment of learning in the water maze, which Richard had developed (Morris et al., 1986). A few years later, the lab of Philippe Ascher in Paris discovered that the NMDA receptor channel exhibited a voltage-dependent magnesium blockade and was also permeable to calcium (Johnson and Ascher, 1990). These properties provided a mechanism by which the NMDA receptors could play a major role in LTP induction, as it was apparent that these receptors, which are minimally activated by a single presynaptic stimulation, are activated during the trains of high frequency stimulation used to elicit LTP, and then produce the influx of calcium required for LTP induction. Several additional pieces of evidence contributed to revive the hypothesis that calpain activation could participate in LTP and in learning. Thus, activation of NMDA receptors resulted in calpain-mediated spectrin degradation (Seubert et al., 1988), which strongly supported the notion that activation of NMDA receptors during LTP induction could lead to calpain activation and spectrin truncation, which could then provide the basis for the structural reorganization of dendritic spines (Baudry and Lynch, 2001). The only missing piece was still the link between calpain activation and the potential increase in the number of AMPA receptors, which are responsible for the generation of synaptic responses.

## 4 The USC period

In the late 80s, the University of Southern California (USC) in Los Angeles charged Bill Wagner and Bill McClure to create a multidisciplinary program to study the brain, the Neural, Information and Behavioral Science (NIBS) program. This was at the times quite a revolutionary idea, as it was combining biological sciences, computer sciences and psychology to make breakthrough contributions to the understanding of brain systems and functions. They were help by a donation from the Hedco Foundation, which allowed the construction of a new building on the Park Campus at USC, the Hedco Neuroscience Building (HNB). Bill Wagner and Bill McClure were able to recruit Richard Thompson who was then at Stanford to be the director of NIBS. Richard Thompson recruited a number of talented scientists to move into the Keck Neuroscience Building, and although he could not recruit Gary Lynch, he did recruit Michel Baudry, who moved into the HNB in the Fall of 1989. By then, it became possible to study the AMPA receptors by using 3H-AMPA as a ligand in the widely used binding assays, either with isolated synaptic membranes or with tissue sections and autoradiography. In a very successful collaboration between the laboratories of Richard Thompson and Michel Baudry, a series of studies concluded that LTP induction *in vivo* was associated with increased ^3^H-AMPA binding in hippocampus (Tocco et al., 1992a,b), with the increase in binding correlated with the amplitude of LTP (Maren et al., 1993). These results provided strong support for the original hypothesis that LTP was associated with an increase in glutamate receptors, at least for the AMPA receptors.

The idea that calpain was somehow involved in the regulation of glutamate receptors was never abandoned, and we discovered that, in fact, calpain was able to cleave both the AMPA and the NMDA receptors, as well as several postsynaptic proteins regulating the anchoring of these receptors to the postsynaptic membranes. These later findings provided for a novel mechanism linking calpain activation with changes in postsynaptic AMPA receptors. This notion was developed in a manuscript by Baudry and Lynch where they revisited the original Science hypothesis 20 years later (Baudry and Lynch, 2001). In this review, they proposed that calpain-mediated cleavage of spectrin and various anchoring proteins was facilitating the insertion of new AMPA receptors in the postsynaptic density. This mechanism also provided an explanation for the transformation of silent synapses, which they postulated the existence 10 years earlier (Lynch and Baudry, 1991). In addition, calpain-mediated AMPA receptor truncation was postulated to be involved in the removal of AMPA receptors from the postsynaptic membranes, which could represent a mechanism for either LTP reversal, which has been found to take place under various experimental conditions (Larson et al., 1993; Norris et al., 1996) or for the induction of long-term depression (Dudek and Bear, 1992). The notion that calpain was involved in receptor membrane trafficking was supported by additional findings (Wu and Lynch, 2006), including the calpain-mediated cleavage of stargazin (Yu et al., 2011).

Unfortunately, a new finding gave what looked like a fatal blow to the calpain hypothesis of learning and memory. Dr. Chishti had generated a global calpain-1 knock-out (ko) mouse to study the role of calpain in blood cells (Kuchay et al., 2012; Wieschhaus et al., 2012). Dr. Baudry eagerly asked him if he could test the calpain hypothesis using these mice and Dr. Chishti provided the lab with 6 wild-type and 6 calpain-1 ko mice, which were already relatively old (6–8 months). While the expectation was that these mice would exhibit impairment in learning and in LTP, these mice appeared to be perfectly normal (Grammer et al., 2005). The only positive finding was that there was a slight impairment of paired-pulse facilitation at the CA3-CA1 synapses, suggesting a potential involvement of calpain-1 in the regulation of glutamate release. Another possibility that was suggested by these results was that it was not calpain-1 that was involved in synaptic plasticity and learning, but rather the other significant calpain isoform present in the brain, calpain-2. As will be discussed later, this turned out not to be the case.

In parallel to these studies on the potential roles of calpain in synaptic plasticity, a number of studies indicated that calpain could be involved in neurodegeneration (Siman, 1992; Nixon, 2000; Araujo and Carvalho, 2005; Artal-Sanz and Tavernarakis, 2005). In fact, these studies led Gary Lynch and several of his colleagues at UCI to start a company, Cortex Pharmaceuticals, to develop calpain inhibitors for the treatment of neurodegenerative disorders. In this regard, the Baudry lab made an important finding and showed that calpain-mediated truncation of another glutamate receptors, the mGluR1a, resulted in switching the neuroprotective function of this receptor to a neurodegenerative function and represented a key step in excitotoxicity (Xu et al., 2007). In particular, they showed that preventing calpain-mediated truncation of mGLluR1a by using a small decoy peptide targeting the truncation site, provided protection against excitotoxicity. Several laboratories had already showed that calpain was activated following kainate-induced seizure activity (Siman and Noszek, 1988; Najm et al., 1992; Bi et al., 1996) and it had been proposed that such activation played a critical role in the pathological consequences of seizures (Araujo et al., 2004; Araujo et al., 2008). After a few years, the calpain inhibitor program at Cortex Pharmaceuticals was replaced by a program directed at developing a new class of molecules, positive AMPA receptor modulators called Ampakines, for the treatment of a variety of neurological and neuropsychiatric disorders. The calpain inhibitor program was then acquired by Alkermes, and Alkermes scientists confirmed that calpain was abnormally activated during cellular events leading to neuronal damage, such as in stroke, global ischemia and traumatic brain injury (TBI) (Bartus et al., 1995). They also pointed out that prolonged calpain activation could play a critical role in chronic neurodegenerative disorders, such as Alzheimer’s disease, Parkinson’s Disease and Amyotrophic Lateral Sclerosis (ALS) (Bartus, 1997). However, Alkermes did not pursue the clinical development of their calpain inhibitors.

A major advance in the field of calpain was provided by findings from several laboratories. In 2004, the laboratory of Alan Wells reported that EGF exerted its effect on epithelial cell motility in part by activating calpain-2 as a result of MAP kinase-mediated phosphorylation (Glading et al., 2004). The Baudry lab was sufficiently intrigued by this finding that they started exploring the possibility that EGF but also BDNF could activate calpain-2 by a similar mechanism. Their results demonstrated that both BDNF and EGF could activate calpain-2 through ERK-mediated phosphorylation (Zadran et al., 2010b). This was a major finding as it eliminated one of the critical roadblocks to explain how calpain-2 could be activated under physiological conditions, since it requires almost millimolar calcium concentration for *in vitro* activation. These results again raised the possibility that it was calpain-2 that played a key role in LTP and in learning and memory (Zadran et al., 2010a).

## 5 The WesternU period

In 2006, Dr. Bi moved from UCI where she was assistant professor to the College of Osteopathic of the Pacific (COMP) at Western University of Health Sciences (WesternU) in Pomona, CA. In turn, Dr. Baudry was recruited by WesternU to become the Dean of the Graduate College of Biomedical Sciences in 2012. Within a few years, our collaboration resulted in major findings, which both validated and greatly expanded the original calpain hypothesis of learning and memory. One of the first experiments which validated the original hypothesis was to test the effects of a calpain inhibitor on LTP when applied either before, shortly after or 1 h after LTP induction elicited by what had been found by the Lynch’s lab to be the optimal protocol, i.e., short bursts of high frequency stimulation repeated at the theta frequency (typically, 10 bursts of 5 pulses at 100 Hz delivered at 5 Hz, theta burst stimulation (TBS)). The results were remarkable: i) when applied before TBS, the calpain inhibitor totally prevented LTP induction as was found previously (Oliver et al., 1989), ii) when applied 1 h after TBS, the inhibitor has no effect on established LTP, and iii) when applied shortly after TBS (10 min) the inhibitor results in an enhancement of the magnitude of LTP (Wang et al., 2014). Detailed analysis of the underlying mechanism revealed that activation of calpain-1 during the seconds/minutes following TBS resulted in the cleavage of the suprachiasmatic nucleus circadian oscillatory protein (SCOP), a negative ERK regulator, which had previously been implicated in learning and memory (Shimizu et al., 2007), thus activating ERK and the signaling pathways leading to LTP. However, SCOP is rapidly regenerated by mTOR-mediated local protein synthesis, which itself is stimulated by calpain-2-mediated degradation of PTEN, an inhibitor of mTOR. In this model, calpain-2 is activated by BDNF-mediated ERK phosphorylation, as BDNF has been shown to be released after TBS (Gartner and Staiger, 2002; Gooney et al., 2002; Lynch et al., 2007; Jourdi et al., 2009; Edelmann et al., 2015).

These results were further validated when the previously negative results obtained with calpain-1 ko mice were revisited with new sets of mice. Under these conditions, we were able to show that deletion of calpain-1 resulted in complete impairment of LTP induction and memory impairment (Wang et al., 2014). The previous negative findings in these mice were explained when we discovered that various types of electrical stimulation used to induce LTP used different signaling pathways for producing the similar increase in synaptic efficacy (Zhu et al., 2015). Likewise, subtle differences in training protocols also accounted for the lack of learning impairment we previously observed with the calpain-1 ko mice. The conclusions from these studies indicated that calpain-1 and calpain-2 play opposite functions in synaptic plasticity and learning and memory (Baudry and Bi, 2016). This calpain-1 activation is required for triggering LTP and initiating the memory process, while calpain-2 activation limits the extent of LTP and of learning. These conclusions were further validated by assessing first the effects of a selective calpain-2 inhibitor, C2I, on both LTP and learning. The inhibitor enhanced the magnitude of LTP when it is added to hippocampal slices either before or after TBS (Wang et al., 2014). When administered to mice 1 h before training the inhibitor elicited facilitation of learning at low doses and inhibited learning at high doses (Liu et al., 2016b). The ratio of the doses producing inhibition of learning over the doses resulting in facilitation of learning matched the ratio of the IC50s for inhibiting calpain-1 over calpain-2. As calpain-2 deletion is embryonically lethal, we generated a conditional calpain-2 ko mice with calpain-2 deletion in excitatory neurons of the forebrain. These mice also exhibited enhanced learning in both the novel object recognition task and the fear conditioning task (Liu et al., 2016b).

In addition to playing a significant role in LTP, calpain-1 was also found to participate in various forms of long-term depression (LTD), and in particular in mGluR-dependent LTD. Thus, LTD was found to be inhibited by a calpain inhibitor (Hrabetova and Sacktor, 1996), and mGluR-dependent LTD was impaired in field CA1 of calpain-1 ko mice (Zhu et al., 2017). The notion that various proteases play critical roles in synaptic plasticity has recently been reviewed (Salazar et al., 2016).

In view of the opposite roles of calpain-1 and calpain-2 in synaptic plasticity, we started to study whether calpain-1 and calpain-2 could also play opposite functions in neuroprotection/neurodegeneration. We already had some indications that this could be the case, as it had recently been shown that the NMDA receptors could also exhibit both neuroprotective and neurodegenerative properties depending on their subcellular localization (Hardingham, 2009). Thus, the synaptic receptors, consisting mostly of NR2A subunits were neuroprotective while the extra-synaptic NMDA receptors consisting of NR2B subunits were linked to neurodegenerative signaling pathways. We first determined that calpain-1 could co-precipitate with NR2A receptors and its stimulation in cultured neurons resulted in neuroprotection against starvation and oxidative stress-induced damage. This effect was mediated by calpain-1-induced cleavage and inactivation of the phosphatase PHLPP1 and the activation of the neuroprotective pathway comprising Akt and ERK1. In contrast, activation of extrasynaptic NMDA receptors stimulated calpain-2, which promoted the activation of another phosphatase, STEP, and neuronal death. These *in vitro* results were further validated in a series of experiments using different animal models of acute neurodegeneration (Wang et al., 2013).

We first analyzed the roles of calpain-1 and calpain-2 in two models of acute degeneration of retinal ganglion cells produced by direct intraocular NMDA injection or increase in intraocular pressure (Wang et al., 2016b,2018a). In both models we determined that calpain-1 and calpain-2 were activated but with very different time-courses. Calpain-1 was rapidly and transiently activated, while calpain-2 activation was delayed and prolonged (Wang et al., 2016b). Furthermore, ganglion cell damage was exacerbated in calpain-1 ko mice, and either systemic or intraocular administration of a selective calpain-2 inhibitor following the return to normal of intraocular pressure, provided complete protection of the retinal ganglion cells. It also prevented the development of blindness, which follows neuronal degeneration (Wang et al., 2016b).

We were contacted by a British neurologist, Dr. Holden, who was following a patient with a null mutation of calpain-1 and who exhibited severe cerebellar ataxia. It had also been reported by the Russel Terrier dog, which presents with a null mutation of calpain-1, also exhibits cerebellar ataxia (Forman et al., 2013). We carefully analyzed the gait properties of our calpain-1 ko mice and found that they also exhibited cerebellar ataxia (Wang et al., 2016a). Detailed studies of the mechanisms underlying this effect revealed that the lack of calpain-1 results in enhanced neuronal apoptosis during the postnatal period. It also results in a lack of maturation of dendritic spines throughout the brain. These effects were the consequences of the lack of calpain-1-induced degradation of PHLPP1 and the resulting Akt activation during the postnatal period. As a result, there was a decrease in the number of cerebellar granule cells and an impairment of synaptic transmission in the cerebellum (Heysieattalab et al., 2020). These effects were prevented by the administration during the postnatal period of an Akt activator, bisperoxovanadium, or by crossing calpain-1 KO mice with PHLPP1 KO mice (Liu et al., 2016a). Since then, a number of families with mutations in calpain-1 have been identified and they all exhibit spinocerebellar ataxia and cognitive impairments (Gan-Or and Rouleau, 2016; Cotti Piccinelli et al., 2019; Peng et al., 2019).

As calpain activation had previously reported to participate in neuronal degeneration in traumatic brain injury (TBI), we investigated whether calpain-1 and calpain-2 also played opposite functions in a mouse model of TBI, the controlled cortical impact model (Wang et al., 2018b). This is a model of severe TBI, as it consists of opening the skull and hitting the brain with a small piston, which produces a very reproducible lesion. The results we obtained in this model completely validated our previous conclusions (Wang et al., 2018b). Again calpain-1 was rapidly but transiently activated in the cortex surrounding the lesion site, while calpain-2 activation was delayed and prolonged, lasting several days. The extent of lesion was larger in the calpain-1 ko mice and was reduced in the conditional calpain-2 ko mice (Wang et al., 2018a). Injection of a selective calpain-2 inhibitor 1 h after the trauma significantly reduced neuronal death in the days following the insult but had a non-significant effect 1 month later. In contrast, daily injections of the inhibitor for 7 days after TBI produced a highly significant reduction in the lesion volume. These results confirmed that calpain-2 activation is very prolonged after TBI and the inhibitor needs to be administered during all this period to minimize neuronal death (Baudry et al., 2023).

We found similar results in a model of repeated mild concussion, in which non-anesthetized mice receive repeated blows to the head for several days (Wang et al., 2020). These mice develop a pathology very similar to what is observed in humans suffering from chronic traumatic encephalopathy, i.e., brain inflammation, neuronal damage, translocation of TDP-43, and cognitive impairment. All these pathological features were prevented by a simultaneous treatment with a selective calpain-2 inhibitor of in the conditional calpain-2 ko mice.

Finally, we also found a similar pattern for the functions of calpain-1 and calpain-2 following status epilepticus elicited by repeated injections of low doses of kainate in mice (Seinfeld et al., 2016). In this case, the pattern of activation for calpain-1 and calpain-2 was quite different. While calpain-2 activation was widespread in the dentate gyrus and the hippocampal pyramidal cells, calpain-1 activation was restricted to a small population of parvalbumin-containing interneurons. In this case as well, daily administration of a selective calpain-2 inhibitor for 7 days after seizures completely prevented the pathological manifestations produced by seizures, i.e., brain inflammation, neuronal death, and cognitive impairment. Similar results were found in the conditional calpain-2 ko mice (Wang et al., 2021).

All these studies provide a coherent picture regarding the opposite functions of calpain-1 and calpain-2 in the brain. Thus, calpain-1 activation is required for triggering certain forms of synaptic plasticity and of learning and memory. On the other hand, calpain-2 activation limits the extent of synaptic plasticity and of learning. Calpain-1 activation is neuroprotective and lack of calpain-1 or its inhibition results in exacerbated neuronal damage and potentially neurodegenerative disorders. Prolonged calpain-2 activation takes place following various types of acute neuronal injury, including concussion and seizures, and is critically involved for the development of neuronal damage. Consequently, a selective calpain-2 inhibitor produces two effects: i) it facilitates learning and ii) it is neuroprotective. Through a program funded by the DoD, we have now identified a lead clinical candidate, which exhibits a potent and selective inhibition of human calpain-2, referred to as NA-184, and are planning clinical trial with this molecule in the near future (Baudry et al., 2023). It will be particularly interesting to test whether this molecule also facilitates learning in human subjects.

## 6 Conclusion

**FIGURE 1:**
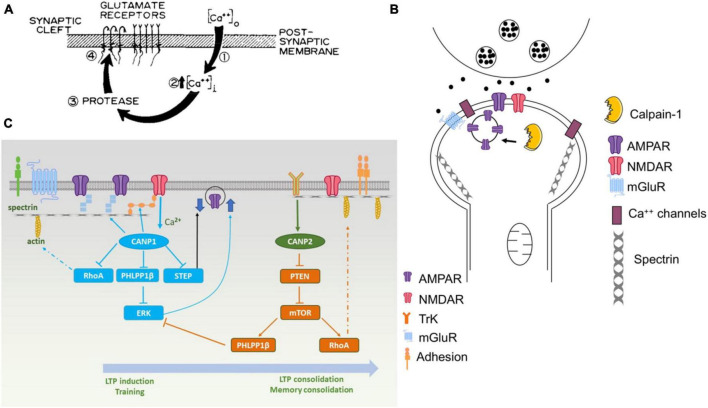
Various versions of the original hypothesis on the role of calpain in learning and memory. **(A)** First version of the model published in Baudry and Lynch (1980a). The protease postulated in this model was rapidly identified as being calpain. **(B)** Revised model published in 2001 [adapted from Figure 4 from Baudry and Lynch (2001)]. **(C)** Recent model published in Baudry and Bi (2016). Calpain-1 is downstream of synaptic NMDA receptors and, when activated, cleaves several proteins participating in LTP induction and memory formation. On the other hand, calpain-2 is activated by TrkB-mediated ERK activation and phosphorylation and stimulate local protein synthesis through the mTOR pathway.

It took over 40 years to validate and refine the original hypothesis that calpain activation played a critical role in learning and memory. Figure 1 illustrates the various versions of this hypothesis over the years and how more details regarding the underlying mechanisms were added. In particular, we have now identified several targets of calpain-1 and calpain-2 that account for the opposite roles of these 2 calpain isoforms in synaptic plasticity and learning and memory. Perhaps future research will find more mechanistic pathways linking calpain to cognitive functions. The identification and development of a selective calpain-2 inhibitor will allow to test, at least the inhibitory role of calpain-2 in learning, in humans in the very near future. The stimulatory role of calpain-1 in learning and memory is already indicated by studies from patients with deletion/mutations of calpain-1, which suggest that these patients do exhibit cognitive impairment (Mereaux et al., 2021; Alecu et al., 2022). It would be of interest to develop a selective calpain-1 activator, which would have the dual benefit of stimulating learning and memory and being neuroprotective.

While the studies discussed in this review have focused on the roles of calpain in learning and memory and in neurodegeneration, it is clear that they provide only a partial representation of the more complex description of both processes. In particular, the protein kinase CamKII has long been recognized as being a “memory molecule” (Lisman et al., 2002; Yasuda et al., 2022). Although still very controversial, another protein kinase, PKMzeta has also been proposed to underlie the maintenance of memories in the brain (Sacktor, 2012; Glanzman, 2013; Patel and Zamani, 2021). As we argued in a previous review (Baudry and Bi, 2013), learning and memory evolves from basic cell biological mechanisms, which were involved in cell motility and other basic cellular functions, and it is therefore not surprising that a multitude of molecular events participate in this process, which is essential for survival. A full description and integration of all these processes in a unitary model of learning and memory still needs to be proposed.

## Author contributions

MB: Conceptualization, Writing – original draft. XB: Conceptualization, Writing – original draft.
